# Neck circumference and cardiometabolic risk in children and adolescents: the moderator role of cardiorespiratory fitness

**DOI:** 10.1186/s12887-021-02696-y

**Published:** 2021-05-17

**Authors:** Ana Paula Sehn, Caroline Brand, Letícia Welser, Anelise Reis Gaya, Cesar Agostinis-Sobrinho, Carlos Cristi-Montero, Elza Daniel de Mello, Cézane Priscila Reuter

**Affiliations:** 1 Graduate Program in Health Promotion, University of Santa Cruz do Sul (UNISC), Independência Av, 2293 – Universitário, Santa Cruz do Sul, RS 96815-900 Brazil; 2 Graduate Program in Human Movement Sciences, Federal University of Rio Grande do Sul (UFRGS), Porto Alegre, RS Brazil; 3 Faculty of Health Sciences, Klaipeda University, Klaipeda, Lithuania; 4 IRyS Group, Physical Education School, Pontificia Universidad Católica de Valparaíso, Valparaíso, Chile; 5 Graduate Program in Child & Adolescent Health, Federal University of Rio Grande do Sul (UFRGS), Porto Alegre, RS Brazil

**Keywords:** Anthropometry, Pediatrics, Physical fitness, Obesity, Metabolic syndrome

## Abstract

**Background:**

The increased incidence of cardiometabolic risk factors has become a public health issue, especially in childhood and adolescence. Thus, early identification is essential to avoid or reduce future complications in adulthood. In this sense, the present study aimed to verify the influence of cardiorespiratory fitness (CRF) as a moderator in the association between neck circumference (NC) and cardiometabolic risk in children and adolescents.

**Methods:**

Cross-sectional study that included 2418 randomly selected children and adolescents (52.5% girls), aged 6 to 17 years old. Anthropometric measurements, such as NC and body mass index (BMI), and CRF was measured by the six-minute running/walking test, as well as cardiometabolic risk (systolic blood pressure, glucose, HDL-C, and triglycerides), were assessed.

**Results:**

For all age groups, NC showed a negative relationship with CRF. A significant interaction term was found for CRF x NC with cardiometabolic risk for children (6 to 9 years old), early adolescents (10 to 12 years old), and middle adolescents (13 to 17 years old). It was found that children who accomplished more than 1092.49 m in CRF test were protected against cardiometabolic risk when considering NC. In adolescents, protection against cardiometabolic risk was found when the CRF test was completed above 1424.14 m and 1471.87 m (early and middle stage, respectively).

**Conclusions:**

CRF is inversely associated with NC and acts as a moderator in the relationship between NC and cardiometabolic risk in children and adolescents. Therefore, this detrimental health impact linked to fatness might be attenuated by improving CRF levels.

## Background

The increased incidence of cardiometabolic risk factors such as obesity, and physical inactivity, has become a public health issue, especially in childhood and adolescence worldwide [1, 2]. Thus, early identification is essential for generating strategies and monitoring public health progress to avoid or reduce adulthood complications [3, 4].

In this sense, overweight and obesity are strongly associated with the development of cardiometabolic diseases [5–7]. Obesity plays an essential role in metabolic disorders, since leads to a low-grade chronic inflammation increasing the concentration of proinflammatory proteins, such as neutrophils, monocytes, and C-reactive protein [8]. In addition, adiposity influence the development of dyslipidemia, increasing liver adipocytes [9], as well as in blood pressure levels [10], due to an increase of inflammatory markers, such as interleukin-6 that can cause an increase in C-reactive protein. This increase of C-reactive protein is associated with less nitric oxide that causes lower vasodilation dependent on the endothelium, and consequently enhances the risk of developing cardiometabolic diseases [11]. Therefore, adipose tissue is a complex and highly active metabolic endocrine organ [12].

Anthropometric measures, such as body mass index (BMI) and waist circumference, are the most common tools to identify overweight and obesity in children and adolescents [13, 14]. Also, there is evidence indicating that neck circumference (NC) presents a good correlation with adiposity, BMI, and waist circumference [15, 16], demonstrating accuracy of 77.2% for diagnostic of obesity [17]. Thus, it is considered an accurate tool for screening and assessing overweight and obesity in different age groups [15, 18–20]. This measure is simple, practical, and low-cost, as well as it has been suggested as a better alternative than waist circumference [21]. Moreover, NC demonstrates a similar relationship that other adiposity measures with cardiometabolic diseases in childhood [22]. A recent study developed with Brazilian adolescents showed that NC presented good sensitivity and specificity to identify excess weight [23]. Thus, NC can be used to identify cardiometabolic diseases in the pediatric population [3, 24–26].

In contrast, cardiorespiratory fitness (CRF) has an important role in improving cardiometabolic health, as it is known that when individuals present high CRF levels, lower is their cardiometabolic risk profile [27, 28]. Evidence indicates that direct measures are more recommended for evaluating CRF related to cardiovascular disease [29], however, indirect measures, such as field tests are also considered as a useful alternative [30–32]. The six-minute running/walking test is commonly used for the evaluation of Brazilian schoolchildren, due to low operating costs, ease of application and the opportunity to evaluate a large number of individuals simultaneously [33, 34]. It is also useful for tracking children and adolescents at risk of developing negative health outcomes [35, 36]. The possible mechanisms by which CRF exerts these benefits are associated to a reduction of low-grade inflammation due to the high utilization of nutrients as a source of energy, mitochondrial biogenesis, reduction of visceral fat and blood lipid profile [37].

CRF seems to be inversely related to NC [24], although this relationship is under-explored in the literature, the association between NC and cardiometabolic risk is already established. However, there is no evidence indicating the moderator role of CRF in the relationship between these variables, as well as the point at which the level of CRF begins to protect against the development of cardiometabolic diseases. Due to the high prevalence of fatness at the global level and their close relationship with cardiometabolic diseases is relevant to explore factors that could mitigate this unfavorable scenario on the children and adolescent’s health status. Taking these aspects into consideration we hypothesized that high CRF levels could counteract the deleterious influence of NC in cardiometabolic risk. In this sense, the present study aimed to verify the association between CRF and NC and the moderator role of CRF in the relationship between NC and cardiometabolic risk in children and adolescents.

## Methods

This is a cross-sectional study developed with 2418 children and adolescents aged 6 to 17 years old, from private and public schools from the city of Santa Cruz do Sul-RS, Brazil. The sample was randomly selected. Since 2004, the same schools have participated in this research called “Schoolchildren’s Health” to form a cohort. For this, a survey was carried out in the city to obtain the number of schools (*n* = 50) and enrolled students (*n* = 17,688). The population density of schoolchildren in all regions of the city, including public (municipal and state) and private schools, was considered to perform the sample size calculation. This study was approved by the Human Research Ethics Committee of the University of Santa Cruz do Sul (UNISC) (number 1.498.305) and followed the resolution 466/2012 of the National Council of Health in Brazil.

The sample size calculation for the present study was performed using the G*Power 3.1 program (Heinrich-Heine-Universität - Düsseldorf, Germany). According to Faul, Erdfelder, Buchner, & Lang [38], the most appropriate statistical test to use is multiple linear regression. As reference parameters it was used: test power (1 - β) = 0.95, effect size (f^2^) = 0.02 and significance level α = 0.05, estimating a minimum sample of 995 individuals.

### Measures

NC was measured with the most prominent portion of the thyroid cartilage taken as a reference, with plastic tape, and accuracy of 0.1 cm. Weight and height were assessed through an anthropometric scale with a coupled stadiometer (Filizola®), while the BMI was calculated through the formula: weight/height^2^. CRF was assessed by the six-minute running/walking test performed on an athletics track, in which the individual should run or walk performing the greatest number of laps. The evaluator noted, and later, added the distance covered by the individuals. The result was obtained in meters. This test followed the protocols by *Projeto Esporte Brasil* [39].

Sex and ethnicity were obtained through a self-reported questionnaire, in which they should tick one of the following options: sex (female and male) and ethnicity (white, black, brown/mulatto, indigenous and yellow). The criteria proposed by Tanner, established maturation stages, considering figures of breast development in girls and testicular development in boys. For this, the evaluator showed the figures with the different stages, and the individual should indicate which stage he/she was in at the current moment. Five stages were considered, which were subsequently categorized into four categories: prepubertal (stage I), initial development (stage II), continuous maturation (stages III and IV), and matured (stage V) [40]. Blood pressure was measured using the auscultatory method with a sphygmomanometer, a stethoscope on the left arm and, a cuff appropriate to the individual’s brachial circumference. It was recommended that the individual remains at rest for 5 min. Two measurements were made, considering the lowest systolic blood pressure. Biochemical parameters (glucose, high-density lipoprotein cholesterol (HDL-C) and triglycerides) were assessed by collecting blood samples after 12 h of fasting. Analyzes were performed on the Miura 200 automated equipment (ISE, Rome, Italy) using serum samples and commercial Kovalent / DiaSys kits (DiaSys Diagnostic Systems, Germany).

A z-score was used to establish cardiometabolic risk, in which the individual z-score of the following variables is added: systolic blood pressure, glucose, HDL-C, and triglycerides. The z-score was calculated using the following formula: z-score ([value of a continuous variable - cutoff points] / standard deviation). The cutoff points and standard deviation were used as proposed by Stavnsbo et al. [41]. HDL-C has an inverse relationship with cardiometabolic risk, so it was multiplied by − 1. Age and sex were also considered for the calculation of the individual z-score of the variables.

### Statistical analysis

Descriptive statistics were used to characterize the sample. The mean and standard deviation were used for continuous variables and relative and absolute frequency for categorical variables. ANOVA and chi-square were applied to compare groups according to age classification. Generalized linear models were used to verify the direct relationship among CRF, NC and cardiometabolic risk.

Linear regression models were used to test moderation analyzes through the PROCESS macro for the Statistical Package for Social Sciences version 23.0 (SPSS; IBM Corp, Armonk, NY, USA). Children and adolescents present different characteristics of development, thus for a better explanation of the the results within the age groups, the analyzes were divided in children (6 to 9 years), early adolescents (10 to 12 years old), and middle adolescents (13 to 17 years old). The following models have been tested: interaction CRF x NC and cardiometabolic risk in children (6 to 9 years old (Model 1); in early adolescents (10 to 12 years old (Model 2); and in middle adolescents (13 to 17 years old (Model 3). Variables that presented interaction were tested according to the Johnson-Newman technique to establish the moderation point, in which CRF was classified according to tertiles. All analyses were adjusted for sex, ethnicity, and maturational stage. The level of statistical significance was established as *p* < 0.05.

## Results

Participant’s characteristics are presented in Table 1 according to age classification. Adolescents from 13 to 17 years old showed higher mean values of NC, CRF, systolic blood pressure, glucose, triglycerides, and cardiometabolic risk in comparison with other age groups. In addition, children from 6 to 9 years old, showed higher mean values of HDL-C were observed compared early and middle adolescence group.

**Table 1:** Participant’s characteristics

Characteristics	Mean (SD)		
	Children (***n*** = 613)	Early Adolescence group (***n*** = 876)	Middle Adolescence group (***n*** = 929)
Age (years)	8.04 (0.93)	11.03 (0.84)	14.40 (1.26)^a^
Weight (kg)	32.79 (8.65)	45.27 (12.14)	58.24 (12.76)^a^
Height (m)	1.33 (0.08)	1.50 (0.08)	1.63 (0.09)^a^
Body mass index (kg/m^2^)	18.35 (3.57)	20.08 (4.42)	21.82 (3.79)^a^
Neck circumference (cm)	28.13 (2.39)	30.19 (2.84)	32.58 (2.81)^a^
Cardiorespiratory fitness (m)	832.17 (141.92)	860.64 (171.42)	950.34 (223.95)^a^
Systolic blood pressure (mmHg)	97.01 (11.46)	103.62 (11.86)	111.55 (11.88)^a^
Glucose (mmol/L)	4.75 (0.36)	4.92 (0.37)	4.92 (0.39)^a^
High-density lipoprotein cholesterol (mmol/L)	1.75 (0.28)	1.52 (0.27)	1.43 (0.26)^a^
Triglycerides (mmol/L)	0.74 (0.33)	0.80 (0.35)	0.81 (0.37)^a^
Cardiometabolic risk (z-score)	−0.38 (2.34)	−0.32 (2.40)	−0.09 (2.24)^a^
**n (%)**			
**Sex**			
Male	291 (47.5)	367 (41.9)	413 (44.5)
Feminine	322 (52.5)	509 (58.1)	516 (55.5)
**Cardiorespiratory fitness**			
Healthy	451 (73.6)	374 (42.7)	274 (29.5)^a^
Risk	162 (26.4)	502 (57.3)	655 (70.5)
**Maturational stage**			
Pre-pubertal	367 (61.6)*	116 (13.7)	14 (1.5)
Initial development	151 (25.3)	301 (35.6)*	80 (8.7)
Continuous maturation (stage III and IV)	63 (10.6)	399 (47.2)	652 (71.1)^a^
Maturated	15 (2.5)	29 (3.4)	171 (18.6)^a^
**Ethnicity**			
White	510 (83.7)	677 (77.8)	705 (76.5)^a^
Black	40 (6.6)	68 (7.8)	64 (6.9)
Brown/mulatto	59 (9.7)	116 (13.3)	138 (15.0)^a^
Indigenous	0 (0.0)	4 (0.5)	8 (0.9)
Yellow	0 (0.0)	5 (0.6)	7 (0.8)

*SD* Standard deviation, *n* number of participants, *%* percentage. ^a^ANOVA or chi-square for differences between children, early or middle adolescence groups (*p* < 0.05)

For all age groups, NC showed a negative association with CRF, indicating that higher CRF is associated with lower NC (Table 2).

**Table 2:** Association between neck circumference and cardiorespiratory fitness

	CRF		
	β	(95%) CI	p
**Children**			
NC	−17.261	−21.730; −12.791	< 0.001
**Early adolescent group**			
NC	−12.683	−16.517; − 8849	< 0.001
**Middle adolescent group**			
NC	−8.200	−12.970; −3.430	0.001

*CRF* cardiorespiratory fitness, *NC* neck circumference, *CI* confidence interval. All analyses were adjusted for sex, ethnicity, and sexual maturation

In Table 3 is presented the moderator role of CRF in the relationship between NC and cardiometabolic risk. A significant interaction term was found for CRF x NC with cardiometabolic risk in children and adolescents.

**Table 3:** Moderation of cardiorespiratory fitness in the relationship between neck circumference and cardiometabolic risk

	Cardiometabolic risk		
	β	CI (95%)	p
**Children 6 to 9 years old**			
CRF	−0.004	−0.006; −0.003	< 0.001
NC	0.464	0.392; 0.535	< 0.001
CRF x NC	**− 0.001**	**− 0.001; 0.000**	**0.001**
**Early adolescent group**			
CRF	−0.003	−0.004; − 0.002	< 0.001
NC	0.411	0.359; 0.462	< 0.001
CRF x NC	**0.001**	**−0.001; 0.000**	**0.019**
**Middle adolescent group**			
CRF	−0.002	−0.003; − 0.002	< 0.001
NC	0.330	0.276; 0.383	< 0.001
CRF x NC	**0.000**	**−0.001; 0.000**	**0.003**

*CRF* cardiorespiratory fitness, *NC* neck circumference, *CI* confidence interval. All analyses were adjusted for sex, ethnicity, and sexual maturation

**Fig. 1:**
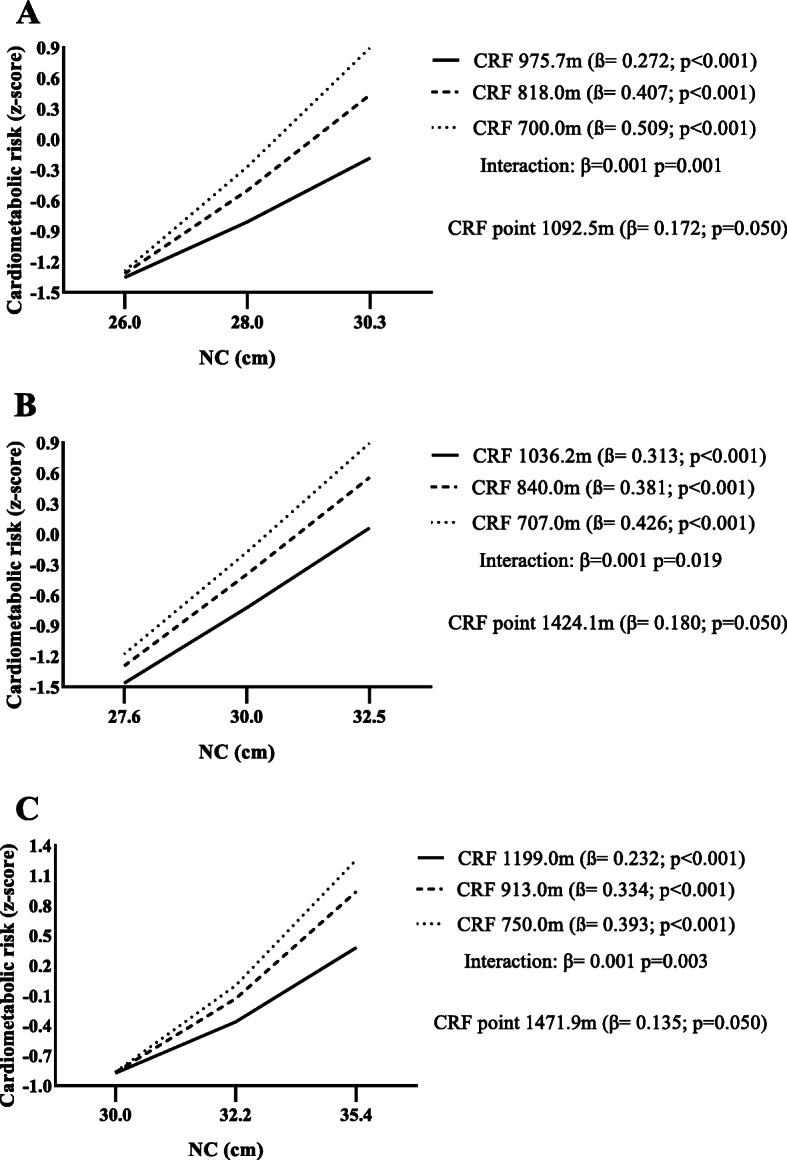
Moderation of cardiorespiratory fitness in the relationship between neck circumference and cardiometabolic risk. **a** children; **b** early adolescents; **c** middle adolescents; CRF: cardiorespiratory fitness; NC: neck circumference

Considering the observed interactions, we intend to establish from which point of CRF there was a protection against cardiometabolic risk. Significant association between NC and cardiometabolic risk were observed in all levels of CRF in children (Fig. 1a), early adolescence (Fig. 1b), and middle adolescence groups (Fig. 1c). Besides, it was found that children who reach more than 1092.5 m in CRF test were protected against cardiometabolic risk when considering NC. In both adolescent groups, protection against cardiometabolic risk was found when the CRF test distance was above 1424.1 m and 1471.9 m, respectively.

## Discussion

Findings of the present study indicate that CRF is inversely associated with NC in children and adolescents of different age groups. Besides, to the best of our knowledge, this is the first study to show that CRF is a moderator in the relationship between NC and cardiometabolic risk factors. Also, the applied analyzes made it possible to establish the level of CRF that children and adolescents must achieve to protect against cardiometabolic risk when NC is considered.

The relationship between CRF and different anthropometric indicators has been widely shown in the literature, which includes an inverse association with BMI, waist circumference, and waist to height ratio [42–46]. However, much less is known about the relation between CRF and NC. Our results indicated that CRF is inversely associated with NC, which is in accordance with a study developed with Spanish children and adolescents [24]. As far as we know, the present study is the only evidence in the literature exploring the link between those variables; nevertheless, CRF was not investigated as a primary outcome but as a moderator. Indeed, CRF is a significant health indicator related to the function of different body systems and organs involved in human health status, such as cardiorespiratory, endocrine-metabolic, and musculoskeletal [47, 48].

Also, it is widely described that NC is associated with some cardiometabolic risk factors in children and adolescents, such as systolic blood pressure, total cholesterol, HDL-C, and clustered cardiovascular diseases risk factors [24–26, 49]. Therefore, these findings indicate that NC is an effective screening measure for identifying metabolic disease risk also at younger ages.

Taking this evidence into consideration, we intend to go further in order to understand the role of CRF in the association between NC and cardiometabolic risk. Results showed that the strength of the relationship between NC and cardiometabolic risk vary according to the level of CRF, highlighting its role as a moderator. This means that in children and adolescents with high CRF levels, there was no longer an association between NC and cardiometabolic risk factors.

In this study, we also indicated the CRF point from which there was no longer an association between NC and cardiometabolic risk. In children, this point was 1092.5 m, early adolescents must achieve 1424.1 m, and in the age group, middle adolescents should accomplish 1471.9 m. The reference values for the CRF test applied in the present study indicate that achieving this goal, children and adolescents from all the age groups considered would be classified in the healthy zone for physical fitness [39]. Therefore, to present protection against cardiometabolic risk, they should achieve significantly high CRF levels. In this context, the role of adiposity must be taken into consideration, once it is known that increased body fat is likely to lead to a decrease in fitness levels in children [50], mainly where body weight is lifted or carried over distance (i.e. running), which may intervene in the tests results [51].

Our study adds to the evidence that NC may be an appropriate marker for cardiometabolic health and could be considered mainly in epidemiological studies, as is a more straightforward and more practical anthropometric parameter, not impeded by clothing, ethical questions, or last meal [21]. More importantly, our data bring new evidence showing the crucial role of CRF, once it can protect against cardiometabolic risk when considering NC. Confirming the idea that appropriate levels CRF, indeed, can mitigate the consequences of body adiposity on cardiometabolic health, although our data showed that only very high CRF levels exert a protection. Still, it is suggested that regardless of body weight, it is important to achieve appropriate CRF levels [44, 52, 53]. This aspect is of great relevance, mainly when considering that the development of different cardiometabolic risk factors has its origin in childhood and can be taken to adulthood [54]. Also, CRF is a modifiable factor that can be enhanced through regular moderate to vigorous physical activity practice [55], highlighting its relevance in the context investigated in the present study.

The cross-sectional data limits us from evaluating the effect of changes in CRF in the analyzed variables. Also, the test used to assess CRF is not a direct measure of aerobic capacity, although it is validated and applied in many investigations [2, 56, 57]. The cutoff points and standard deviation used as proposed by Stavnsbo et al. 2018 does not includes children from Latin American countries [41]. This study was strengthened by the large sample size, investigating moderations according to different age groups and mainly determining the moderation point, since there is no evidence in the literature regarding this aspect.

## Conclusions

CRF is inversely associated with NC and acts as an essential moderator in the relationship between NC and cardiometabolic risk both in children and adolescents. Based on these findings, this detrimental health impact linked to fatness might be attenuated, or even eliminate, by significant improvement in CRF. Thus, this physical fitness marker should be considered in future intervention studies in the pediatric population.

## Data Availability

The database used and analyzed in the present study is not publicly available as its information may compromise the participants’ privacy and consent involved in the research. However, the data are available from the corresponding author (EA), upon request.

## Abbreviations

BMI: Body mass index
CRF: Cardiorespiratory fitness
HDL-C: High-density lipoprotein cholesterol
NC: Neck circumference
UNISC: University of Santa Cruz do Sul
